# An exploration of the oral health beliefs and behaviors of people living with HIV in Mangalore, India: a qualitative study

**DOI:** 10.1186/s12903-021-01549-5

**Published:** 2021-04-30

**Authors:** Anisha Rodrigues, Vijaya Hegde, Adarsh V. Hegde, Suresh G. Shastri, D. N. Ravikumar, Rashmi Rodrigues

**Affiliations:** 1 Department of Prosthodontics, A.J. Institute of Dental Sciences, Mangalore, India; 2 Department of Public Health Dentistry, A.J. Institute of Dental Sciences, Mangalore, India; 3 Care, Support and Treatment, Karnataka State AIDS Prevention Society, Bangalore, India; 4 Department of Community Health, St. John’s National Academy of Health Sciences, Bangalore, India; 5 Global Public Health, Karolinska Institutet, Stockholm, Sweden; 6 Wellcome Trust/DBT India Alliance, Bangalore, India

## Abstract

**Introduction:**

People living with HIV (PLWHIV) have increased oral healthcare needs due to an increase in the prevalence of oral and dental diseases. Their oral health is influenced by psychosocial, behavioral and biologic factors. The aim of this study was to explore and obtain a deeper understanding of the oral health beliefs and behaviors of PLWHIV that could potentially affect their oral health.

**Methods:**

We have used the Health Belief Model (HBM) and qualitative methods using in-depth interviews with 16 PLWHIV. Content analysis of the transcribed data was done. The data was grouped under the constructs of the HBM.

**Findings:**

The perceived susceptibility to oral diseases and awareness on the importance of good oral health was low. Regular tooth brushing and traditional methods for oral hygiene maintenance were considered beneficial. Regular dental visits were not considered important. Psychosocial issues, time and financial constraints were the barriers. Participants believed that information on oral health should be provided by the health providers in hospitals and dental clinics.

**Conclusion:**

The findings on the oral health beliefs and behaviors support the need for education on oral health and preventive healthcare practices among PLWHIV. Oral health promotion should include behavioral change as one of its components.

**Supplementary Information:**

The online version contains supplementary material available at 10.1186/s12903-021-01549-5.

## Introduction

The general well being of people living with HIV (PLWHIV) is greatly influenced by factors such as mental health and socio-demographic characteristics, in addition to their medical conditions [1]. Good oral health is known to positively influence the quality of life of individuals, this is particularly true in case of PLWHIV [2–4]. The inter-relationship between oral health and systemic health is evident especially in patients with chronic diseases, with the signs and symptoms manifested in the oral cavity with varying degree of severity. PLWHIV are more susceptible to oral lesions, the more prevalent being candidiasis, hairy leukoplakia and aphthous ulcers. In addition, they present an increased risk for dental caries, periodontal disease and xerostomia. These oral health issues could affect mastication and swallowing, inadvertently compromising nutrition and possibly adherence to treatment. Furthermore, their immune response could be negatively influenced by poor oral health alluded by the increased levels of immune activation markers found in patients with oral lesions [5]. Though the incidence of HIV-related oral lesions are known to have been reduced from 50% to 10% due to the use of Anti Retroviral Treatment (ART), several studies have shown an increase in the prevalence of dental diseases such as dental caries, periodontitis and tooth wear [6, 7]. The Decayed, Missing, Filled Teeth (DMFT) index in PLWHIV ranges from 8.7 to 18.8, depending on the regional, cultural and demographic differences, along with oral health behaviors and preventive practices, nutrition and access to dental services [10].

In India, studies on the oral health status of PLWHIV have reported varied prevalence rates of oral lesions from 68% to 75%, and a poor dental and periodontal status with the DMFT score of 12.83 [11–13]. A longer duration of ART, depression and other comorbidities have been associated with an increased risk of dental caries [14, 15]. Adding to the burden of dental diseases are the challenges to oral health care mainly due to a lack of oral health awareness, financial concerns and access to care. The findings from the baseline data of a study on oral healthcare in PLWHIV in the US reported that oral health is the persistent unmet need among these individuals, with their oral health deteriorating over time [16].

It is recognized that psychosocial, behavioral and biologic factors play a role in the health of individuals. Behavioral science involves the application of the knowledge of these factors to diagnose and develop interventions for health promotion [1]. Health behaviors and practices arise from and are driven by health beliefs, which in turn influence health outcomes [15]. There are several models that strive to predict and explain health behaviors. The Health Belief Model (HBM) is a psychosocial model and posits that an individual’s health behavior depends on his perception of an illness as a threat to his health and well-being, and that the benefit of a certain action would outweigh the negative effects of inaction [17]. The assumptions of this model are based on 6 constructs: perceived severity, perceived susceptibility, perceived benefits, perceived barriers, self efficacy and cues to action. Therefore, this model can be used to understand the behaviors that have lead to a particular disease or could potentially lead to a disease even before the signs and symptoms develop. As PLWHIV are vulnerable to a plethora of oral diseases, the HBM can be applied to explain and predict the oral health beliefs, behaviors and practices that might influence their oral health status.

There is a lack of contextual information regarding oral health in PLWHIV derived from the patients’ perspective. Furthermore, there is no behavioral research with regard to oral health in the Indian context. Qualitative methods are extensively used in behavioral research to effectively capture the perceptions and experiences of the participants and explain the complexity of behavior. We therefore  used qualitative methods along with the theoretical framework of the HBM as a guide to strengthen our research. The findings from this study could aid in finding strategies and developing preventive interventions that focus more on the individual’s perception of health for effective oral health outcomes. The data obtained from this research will be used to develop a psychometric instrument to assess the oral health behaviors of PLWHIV.

## Methods

This qualitative study is a part of a larger research project, which aims to study the association between oral health behaviors, oral health status and CD4 count in PLWHIV in Mangalore, Karnataka State, India.

Our study sample included PLWHIV who were receiving ART at the ART Centre, Wenlock Hospital, which is a government run district hospital in Mangalore. The healthcare personnel at the ART Centre comprise a medical officer, a nurse, a counselor and a pharmacist. PLWHIV visit the Centre on a monthly basis to refill their antiretroviral medication and for a clinical followup. The PLWHIV visiting the centre were approached and requested to participate in the study by authors AR and VH, who administered the informed consent and conducted in-depth interviews. An interview guide developed for the purpose of this study was used for the interviews, and probes were used to elicit additional details or clarify responses. The interviews were conducted in the local language i.e. either Kannada or Tulu. The interviews lasted for about 20 min, were organised in a quiet room and audio recorded with prior permission of the participants. The sociodemographic details of the participants were recorded. Data saturation was achieved with the 14th participant, after which two more interviews were conducted to confirm saturation. The Health Belief model was used to frame the interview guide with key questions based on its constructs.

## Data analysis

### Data was analyzed using content analysis

The interviews were translated and transcribed to the English language by a native local language speaker proficient in both the local languages and English. The transcripts were then read by the first and second authors and spot checked for consistency with the recordings. AR then familiarized herself with the transcripts and coded them. The text was broken into smaller meaningful units, or codes, which are the specific and meaningful words and ideas expressed by the participants. All relevant text from the transcripts was grouped under these codes. After coding the first few transcripts, the rest of the transcripts were indexed by applying the identified codes. Additional codes were generated as the analysis progressed. The codes from the different transcripts were then grouped under categories. Discussions between the authors enabled triangulation of the results and the reorganization of the codes and categories to ensure that the interviews were interpreted correctly. The codes and categories were then mapped under the five constructs of the HBM, which provided subthemes and themes for the analysis.

## Ethics approval

An approval for the study was obtained from the Karnataka State AIDS Prevention Society, and the Institutional Ethics Committee, A J Institute of Medical Sciences and Research Centre. An informed consent was obtained from the participants prior to enrolment in the study. Verbal and written information regarding the aim, procedure and confidentiality of the study was given to all subjects.

## Findings

**Fig. 1:**
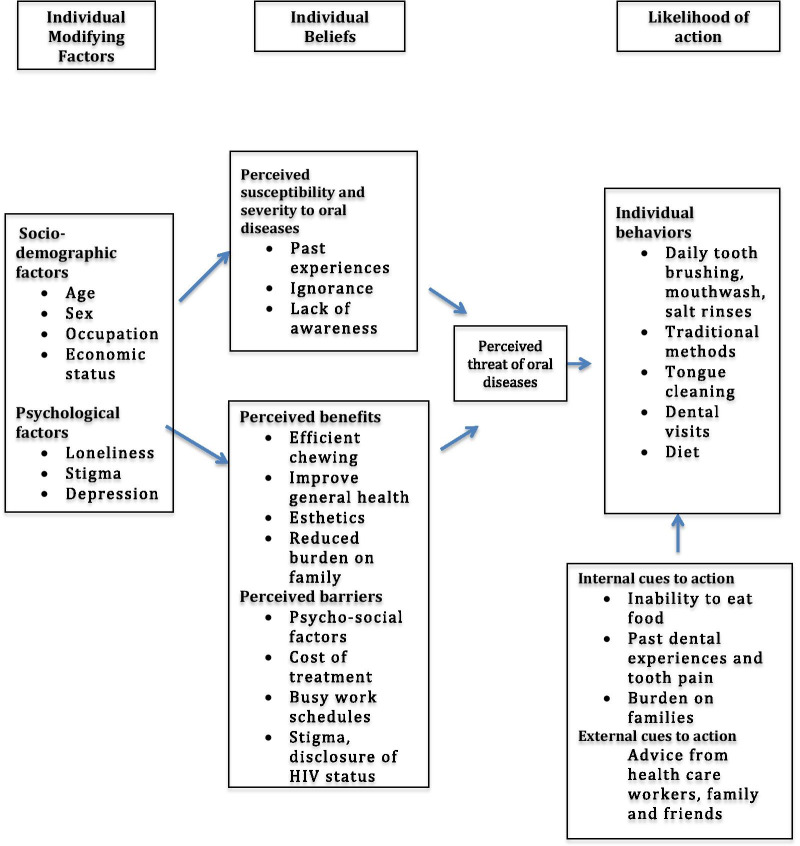
Application of the Health Belief Model to understand the oral health behaviors of PLWHIV

The constructs of the HBM were used to highlight the factors that influenced the oral health behaviors in PLWHIV (Fig. 1). The demographic details of the participants are shown in Table 1.

**Table 1:** Participants’ sociodemographic characteristics

Characteristic	Participants (n = 16)	%
Age	44.93 (± 8.8)	
*Gender* Male Female	9 7	56.25 43.75
*Education* Primary High School Intermediate Post graduate	10 4 1 1	62.5 25 6.25 6.25
*Occupation* Unemployed Retired Unskilled Semiskilled Clerical	2 2 6 5 1	12.5 12.5 37.5 31.25 6.25
*Socioeconomic status* Lower/upper lower Lower Middle	14 2	87.5 12.5
Years since diagnosed with HIV	8.9 (± 7.4)	

Socioeconomic status based on Kuppuswamy’s socio-economic status scale, 2019

### Perceived susceptibility and severity

Most participants believed that HIV would not be the cause of any oral problems as they had them even before being infected. They also did not perceive oral health issues as major problems, even though they had past experiences with mouth ulcers, dental decay and extractions. However, they felt that the future is unpredictable.

On being asked whether she might be susceptible to oral disease due to HIV, one participant replied:

“That I don’t know. It’s with God. (Laughs) it is left to God. It might happen or not. My feeling is that it wont happen.” P_10 (Female)

Participants, therefore considered their susceptibility as low, and were even unaware of the oral health implications of HIV.

However, on probing deeper, some shared their past experiences with oral problems and how it affected them. Tooth pain and ulcers were commonly reported. The ulcers caused pain and burning that lead to an inability to eat food and also a reduced apatite. Some believed that that the tooth pain was due to beeda/tobacco. In many places in India, it is commonly believed that mouth ulcers are caused due to ‘ushna’ (increased ‘heat’) in the body. Therefore, even though a mouth ulcer was the first sign of HIV, it was initially ignored.

“In the mouth I had some ulcer. They said it was because of ‘heat’… I didn’t feel like eating at all. This was before testing. Then they said I should get admitted to the hospital.. Then the mouth problems became less.” P_10 (Female)

These problems prevented them from going to work, and therefore affected their livelihood, as one participant stated:

“I used to earn ten thousand (Rupees) per month, I didn’t go to work for three months. I got ulcer (mouth), I thought during work it might have happened. I had severe pain, burning sensation. I was not even able to have a fruit juice.” P_8 (Female)

Oral lesions had negatively impacted their physical and psychological health that affected their social life.

“I used to have juice. I was worried that I wont be able to eat anything. I thought my life is gone. I used to drink only juice. I became weak. Became very thin. My weight was 39 Kgs. I couldn’t eat. Couldn’t eat anything at all. I didn’t want to talk to anybody. I used to sit inside... Because of mouth problem (ulcers).” P_10 (Female)

### Perceived benefits and self efficacy

The participants were aware of the importance of good oral health. Some believed that poor oral health could affect the general systemic health. The benefits included more efficient chewing, especially hard food, better facial appearance and also a reduced illness related burden on the family. Most patients believed that brushing their teeth twice daily with paste and toothbrush was sufficient to maintain good oral health. Some preferred Ayurvedic toothpaste, as they believe it is made of natural ingredients and therefore safer. Tongue cleaning, mouthwashes and salt rinses were also practiced by a few. One participant felt that the practices that were followed by the older generations such as cleaning teeth with salt, charcoal and cashew/mango leaves are extremely effective.

“Our fathers they had such strong teeth. They all at that time used salt, cashew leaves.. to scrub. Our teeth fell off, not theirs.” P_12 (Female)

Another habit that was practiced was oil pulling. It is an ancient Ayurvedic remedy that is believed to strengthen teeth and improve oral health by swishing a tablespoon of oil for 15–20 min in the mouth. One participant described her family’s practice of oil pulling and its benefits:

“It (oil pulling) prevents disease as much as possible. All toxins from the mouth will come out. Then we drink water. 1 litre. With that all the toxins will go out. We will get taste with food. We will feel like eating food. As soon as we wake up, we put coconut oil in the mouth. Then for half an hour we do oil pulling.” P_3 (Female)

Some participants believed salt has the ability to cure infections. Others felt that chewing paan reduces toothache.

“I brush twice a day. Once in the morning and evening. Then I rinse with salt water because I get throat pain. If I have cold water I get a cold. I make hot water, add salt and rinse my mouth. Then it becomes less.” P_6 (Female)

When questioned about their diet, most participants reported no dietary changes. However, some avoided sweets. Some avoided non-vegetarian food (meat), as they believed it is bad for teeth, but considered fish healthy. Few could not eat hard food as they had unreplaced missing teeth.

There also existed a belief that the body needed to be ‘cooled’ to counter the ‘heat’ to prevent problems.

“I have been told that body should be made cool. I have tender coconut water… so that body doesn’t get 'heat'. They told me to drink juice.. chickoo juice. But I can’t drink too cold also. I get cough. So I have ragi and all. These tablets (ART) are 'heat' no?” P_10 (Female)

#### Benefits of regular dental visits

Tthough participants acknowledged the benefits of good oral health, they believed that regular dental visits and treatment are unnecessary if oral conditions don’t affect them. Some have even pulled out the mobile teeth themselves. Few participants have never visited the dentist in the past.

“No no no… I haven’t (visited the dentist). I didn’t need to remove teeth or had any pain… I never had any problem before. I only come to this doctor here to get tablets. Only if needed I will go to dentist. Only if there is pain we will go isn’t it. (Laughs).” P_5(Male)

The benefits of having a full complement of teeth was perceived to be low, as the partially edentulous state did not affect their daily routines. Though they had missing teeth that needed replacement, some participants preferred to remain partially edentulous. Missing posterior teeth did not affect their looks. Mastication was not a problem as they could use their remaining teeth. There was a perception that as age advances teeth replacement is unnecessary. The other reasons were that they have no children, lived alone, and don’t socialize, so teeth replacement was not required. Moreover, removable dentures were considered cumbersome and fixed dentures expensive.

### Perceived barriers

Psychological barriers like depression and loneliness lead to a lack of motivation and a state of denial of their oral healthcare needs. This, along with a low socioeconomic status and lack of oral health awareness discouraged preventive measures. A female participant said she lived alone, was unaware of where to go, and was therefore dependent on others. She also displayed a low self-confidence and decision-making capability.

Lack of time due to busy work schedules and financial constraints were cited as reasons to defer treatment.

“Yes I feel like cleaning (teeth), but I am not able to go because of work, lot of work… I don’t get time. To keep teeth (denture).. I am ready. But its too much of money.. that’s why I didn’t do. Private and all is very costly.. that’s why I didn’t do it.” P_9 (Male)

Stigma and fear of disclosure were other reasons. There was a fear of a lack of confidentiality that could lead to their stigmatization in the society. To overcome this issue, most participants preferred not to disclose their HIV status to the dentist. They believed that HIV is a personal issue that shouldn’t be disclosed. Some have been informed by their peers not to reveal their HIV status.

“If we tell them (dentists), its like making it public. Next time we will not be able to go there. If we tell we have this problem, won't they tell others or look at us differently.” P_4 (Male)

Regarding the possibility of cross infection, they felt that dentists anyway take precautions. Some believed that because they are on medications, they cannot infect dentists. On the other hand, some felt that it is important to disclose their status as medications can affect the dental treatment. Some revealed their HIV status only when asked.

“When I went to remove my teeth they asked me if I have any problem. I say no. BP, sugar is normal. Then they removed (teeth). They wear gloves isn’t it? They wont have a problem. Most doctors wear gloves and work." P_4 (Male)

### Cues to action

#### Internal cues to action

Many participants had given up deleterious habits like smoking and paan chewing believing that it might adversely affect their general health. The inability to eat food and fear of pain also motivated some of them to maintain good oral health. They didn’t want to be a burden on their children.

“Its is very important, no madam? If there is anything (problem) its difficult to eat. If teeth are not proper, can we eat hard things.. we have to eat soft things, isn’t it? And then if we are not careless about it we can live longer. If we keep it clean only we can protect (teeth). If we don’t keep clean it will go.. because of decay, cavities..if we eat sweets. It is our duty to protect. We use that toothpaste.. Ayurvedic. If tomorrow tooth pain starts, in the night, because of one tooth, the whole thing.. eyes, head and all will have problem.. because of that we are careful.” P_12 (Female)

#### External cues to action

Participants revealed that they received little or no advice on oral health, by health care providers, however friends and family did play a role in motivating them to seek dental treatment. Most were ignorant about oral health, and they believed that information by dentists in their clinics, and doctors at the ART Centre could help create awareness on oral health care. However, as it would be difficult to reach out to every patient, patients themselves could pass on this information to their peers. Posters, handouts or even Short Message Services on the mobile phones regarding oral health in the clinics and hospitals, could increase awareness among patients.

“You can give them information, like a camp (dental) you can do and tell them. It needn’t be them only.. people with disease. It can be any normal person who comes.. you can give them information.. Some go house to house to give information.” P_12 (Female)

## Discussion

The results of our study indicate that the participants had varied oral health beliefs and behaviors. Overall, the participants failed to interrelate oral and systemic health due to which oral health was not prioritized. The unfavorable behaviors were due to a low perception of susceptibility to oral disease and a low level of oral health awareness. Most of our participants believed that their established daily routine of tooth brushing at least once a day, use of tongue cleaners and mouth rinses with water especially after meals would be sufficient to maintain good oral hygiene. Mouthwashes and interdental cleaning aids were rarely used. However, salt rinses were routinely used by the participants as they are widely believed to improve oral health. Though the evidence is conflicting, it is recommended that  salt rinses be used for basic oral health care and maintenance, and cannot replace the conventional toothbrushing and oral hygiene practices [18, 19]. In India, people strongly believe in the indigenous or traditional health practices that use herbs and herbal products for the treatment of various diseases. Ayurvedic medicine is quite popular and is considered safe as it is believed to contain natural ingredients. Mango, cashew leaves and chewing sticks obtained from the stem of neem trees are popular alternatives for toothbrushes. They have been shown to possess anti-microbial properties, effective against plaque and caries, and their extracts used in toothpastes and mouthwashes [20, 21]. However, chewing sticks have caused increased tooth attrition and gingival recession which has been attributed to their improper use [22]. Though there is some evidence of the effectiveness of these products in the general population, further studies need to be conducted to determine the extent of their effectiveness in  PLWHIV.

There is also a strong traditional belief that certain drugs and dietary items such as certain vegetables, fruits, meat and spices produce *heat* as an end result of digestion. This *heat* is called ‘ushna’ and is believed to cause mouth ulcers, skin ailments and digestive problems [23]. Participants in our study believed that the antiretroviral drugs increase ‘ushna’ that causes mouth ulcers, and therefore took measures to counter it by consuming fluids that would ‘cool’ the body. They however made no other modifications to their diet. The perception of the benefits of regular dental visits was deemed very low, and would be considered only if they experienced pain or discomfort as minor issues could be self-managed. Several studies in the general population have also reported a low frequency of dental visits, mainly attributed to a lack of perceived need, cost of treatment and also fear of dental procedures [24, 25].

There were certain barriers to oral health care. Financial barrier has been reported as a major factor for unmet oral health needs even in the general population. Similarly, our participants  highlighted financial constraints as a deterrent to oral health care. The participants in our study belonged to the lower socioeconomic class and a few were retired. They lacked health insurance and were unable to meet the expenses of treatment. Personal factors such as loneliness, worry, low level of self-confidence and dependency on others to travel also acted as barriers. Other studies have reported transportation difficulties, cumbersome administrative procedures, long waits at the dental office and problem focused care-seeking behavior as additional barriers [25–27]. Few participants in our study were ignorant of the location of dental clinics. They seemed unaware of the presence of a dental clinic in the hospital where the ART Centre is located, suggesting the importance of the role of the healthcare workers at the Centre in educating patients and enabling access to dental care.

The fear of disclosure seems to be a universal issue, mainly due to stigma, dentists’ behavior and prejudice, and ignorance towards the clinical implications of the disease [28–30]. Majority of our participants stated that they would not reveal their HIV status to the dentists due to fear of discrimination, refusal of services and confidentiality. Moreover, they felt that there was no risk of transmission due to the general precautions taken by the dentists. This has clear implications for the dentists as several studies have reported inadequate compliance to cross infection control protocols in private dental clinics and dental laboratories [31–34].

It is important that dental professionals understand the perceived barriers and take certain measures so that patients are brought into and maintained under their care. A revision in the dental curriculum is necessary, wherein dental students receive training to enhance social awareness, cultural sensitivity and communication techniques, mainly to curb discriminatory practices and to allay fears and anxiety of the patients. Also, cross infection control should be universally employed for all patients in the dental setting to prevent discrimination. Counseling, sedation could be employed by dentists in their practice to mitigate anxiety. The congenial attitudes of the dental staff who value privacy and offer encouragement and respect serve as motivators for healthy oral health practices [35]. Interventions for PLWHIV should be developed to address dental related fears.

Studies have shown that educational interventions are extremely effective in improving health literacy and promoting preventive oral health behaviors in the target populations such as adolescents, diabetic patients and pregnant women [36–38]. In the current technology driven era, mobile phone applications can serve as attractive means for oral health promotion. A study on the perceptions of an oral health mobile app for oral hygiene routine using animated videos reported that it is a promising tool for patient motivation and education [39]. Further research on development and effectiveness of such technology driven interventions  to include behavior modification for PLWHIV, especially from the lower socioeconomic groups need to be explored.

Our study sample mainly included patients belonging to the lower socioeconomic strata and had a poor perception of oral health. It was observed that the participants belonging to a higher educational background were better equipped with oral health knowledge and had more favorable oral health beliefs and behaviors as compared to the other participants. There exists a negative association between the socioeconomic level and oral and dental disease as people with a higher income and educational background are more likely to regularly brush teeth, use supplementary dental aids and have regular professional dental visits [40]. Socioeconomic inequalities in oral health are known to be associated with the social hierarchy, with oral health deteriorating towards the lower end of the socioeconomic spectrum [41–43].

The findings of our study call for an urgent need for interventions to bring about and sustain positive changes in the beliefs and behaviors towards oral health in PLWHIV. It is recommended that oral health should be integrated with primary health care to facilitate oral health promotion and improve access to oral health services [44]. Health care polices that include attitude and behavior modifications for better oral health should be developed and implemented. Patient education materials in the form of brochures, pamphlets and posters should be made available at ART Centres. Additionally, information could be extended through mobile phones and regular health awareness programs. The professional and lay people involved in providing care to PLWHIV could be instrumental in promoting oral health and should be trained in both general and oral health care, so that they not only create awareness but also refer patients for professional dental care [45]. Initiatives by the government aimed at improving access to oral health care are a prime requisite especially in low and middle income settings, not only for PLWHIV but also for the general population.

## Strengths and limitations

Though we only interviewed patients who visited the ART Centre, and belonged to the lower socioeconomic category, the results are likely to reflect the oral health perceptions of PLWHIV at most ART Centres in the State or country, enabling transferability of the results. Further, the context of the study is extensively described in the introduction, enabling the reader judge the transferability of the results. A detailed description of the study procedures supports the dependability of the results. To ensure credibility of the findings, the researchers discussed the interviews as well as the results at different stages of the analysis and resolved conflicting interpretations of the interviews. This also ensured that the results are credible even though the interviews were not transcribed by a professional agency due to limited funds.

The first author, understanding her role as a researcher and a dental professional, as well as her awareness of the sociocultural background of the participants, supported the contextualization of participant responses as well as their interpretation. Also, the team of researchers involved are from both the public and private healthcare sectors in the country with experience in HIV treatment, policy development, program implementation, as well as research, which strengthens the interpretation of participant responses. Despite this, we consider that further studies may be required to explore the oral health beliefs and behaviors among PLWHIV with different cultural, regional and socioeconomic backgrounds in the Indian context due to its diversity.

## Conclusion

This qualitative study explored the oral health beliefs and behaviors of PLWHIV. The study revealed an alarming lack of oral health awareness and unfavorable oral health behaviors. The findings from this study reveal the utmost necessity of empowering these patients with awareness on the importance of oral health, preventive measures and regular dental visits. There are also implications for health professionals, who need to provide a more holistic approach  to patient management as well as develop a rapport to enhance confidence and allay fears and stigma. Also, oral health care should be made more accessible and affordable by its integration into primary health, especially in low and middle income settings.

## Supplementary Information

**Additional file 1.** Interview guide based on the constructs of the Health Belief Model.

## Data Availability

The data generated from this study are not publicly available, as confidentiality of the participants has to be maintained, but are available from the corresponding author with permission from the Karnataka State AIDS Prevention Society.
