# Supplementary material for: An exploration of the oral health beliefs and behaviors of people living with HIV in Mangalore, India: a qualitative study
Source: BMC Oral Health. 2021 Apr 30;21:222. doi: 10.1186/s12903-021-01549-5 (PMC8086078; doi:10.1186/s12903-021-01549-5)
Supplement: Supplementary file 1 — Additional file 1. Interview guide based on the constructs of the Health Belief Model. [file 12903_2021_1549_MOESM1_ESM.docx]

**Interview guide**

Background

1. What is your age?
2. How long since you have been diagnosed with HIV?
3. Do you have a family?
4. What is your family income per month?
5. What is your educational background?
6. What is your occupation?

**Perceived susceptibility**

1. Could you describe how you feel about your oral health?

Probes:

- What oral health problems have you faced in the past?
- How have you managed those problems?
- What are your concerns due to HIV?

1. Do you know other HIV patients who have had oral health issues?

Probes:

- Could you describe their experiences?
- What is your opinion on being susceptible to similar problems in the future?

**Perceived severity**

1. Could you describe your concerns and worries about your oral health?
2. Could you explain how oral health problems affect your life?

Probes:

- How has it affected you in the past?
- How has it affected your food intake and nutrition?
- How has it affected your work and social life?

**Perceived benefits**

1. Could you describe the benefits of having healthy mouth and teeth?

Probes:

- Need for treatment, tooth replacement
- Regular dental visits

1. Could you talk about your oral health habits that you believe are beneficial for oral health?

Probes:

- Traditional methods
- Diet
- Smoking, paan chewing

**Perceived barriers**

1. Could you explain the issues do you face with your daily oral heath care routine?
2. Could you describe the problems that prevent you from getting regular professional oral health care?

- Personal issues
- Financial issues
- Stigma, fear of disclosure

**Cues to action**

1. What measures would you suggest to increase awareness on oral health for you and other patients?

Probes:

- At the ART centre
- By dental professionals
- Peers

1. How could you be motivated to perform desirable oral healthcare habits?

Probes:

- Compliance
- Regular dental visits
